# Evaluating enrollment and representation in COVID-19 and HIV vaccine clinical trials

**DOI:** 10.3389/fpubh.2024.1411970

**Published:** 2024-07-26

**Authors:** Daisy Lezo Ramirez, Emily Koleske, Omolola Ometoruwa, Jun Bai Park Chang, Urwah Kanwal, Nicholas Morreale, Andres Alberto Avila Paz, Alexandra Tong, Lindsey R. Baden, Amy C. Sherman, Stephen R. Walsh

**Affiliations:** ^1^Division of Infectious Diseases, Brigham and Women’s Hospital, Boston, MA, United States; ^2^Harvard Medical School, Boston, MA, United States

**Keywords:** clinical trial enrollment, diversity, representation, vaccine, HIV, SARS-CoV-2

## Abstract

**Background:**

Vaccine clinical trials should strive to recruit a racially, socioeconomically, and ethnically diverse range of participants to ensure appropriate representation that matches population characteristics. Yet, full inclusion in research is often limited.

**Methods:**

A single-center retrospective study was conducted of adults enrolled at Brigham and Women’s Hospital (Boston, MA) between July 2020 and December 2021. Demographic characteristics, including age, race, ethnicity, ZIP code, and sex assigned at birth, were analyzed from both HIV and COVID-19 vaccine trials during the study period, acknowledging the limitations to representation under these parameters. We compared the educational attainment of vaccine trial participants to residents of the Massachusetts metropolitan area, geocoded participants’ addresses to their census block group, and linked them to reported median household income levels from publicly available data for 2020. Frequency and quartile analyses were carried out, and spatial analyses were performed using ArcGIS Online web-based mapping software (Esri).

**Results:**

A total of 1030 participants from four COVID-19 vaccine trials (*n* = 916 participants) and six HIV vaccine trials (*n* = 114 participants) were included in the analysis. The median age was 49 years (IQR 33–63) and 28 years (IQR 24–34) for the COVID-19 and HIV vaccine trials, respectively. Participants identifying as White were the majority group represented for both the COVID-19 (*n* = 598, 65.3%) and HIV vaccine trials (*n* = 83, 72.8%). Fewer than 25% of participants identified as Hispanic or Latin. Based on ZIP code of residence, the median household income for COVID-19 vaccine clinical trial participants (*n* = 846) was 102,088 USD (IQR = 81,442–126,094). For HIV vaccine clinical trial participants (*n* = 109), the median household income was 101,266 USD (IQR 75,052–108,832).

**Conclusion:**

We described the characteristics of participants enrolled for HIV and COVID-19 vaccine trials at a single center and found similitude in geographical distribution, median incomes, and proportion of underrepresented individuals between the two types of vaccine candidate trials. Further outreach efforts are needed to ensure the inclusion of individuals from lower educational and socioeconomic brackets. In addition, continued and sustained efforts are necessary to ensure inclusion of individuals from diverse racial and ethnic backgrounds.

## Background

The COVID-19 pandemic parallels the early days of the HIV epidemic, with both diseases disproportionally impacting marginalized communities (1). Both began with efforts to identify the causative pathogen but differed between the time to authorization and rollout of effective preventive vaccines (2). Emergency Use Authorization (EUA) for several vaccines against COVID-19 was granted by the US Food and Drug Administration (FDA) less than 1 year after identifying the virus, while for HIV, there are no approved vaccines despite global efforts over the past four decades. Even with the striking impact of COVID-19 on individuals with stark health disparities, including those from communities of color and those that experience multiple forms of social disadvantage, these individuals were not initially well represented in the COVID-19 vaccine trials (3, 4). When sponsors presented the preliminary enrollment reports, Black people comprised only 5% of clinical trial participants despite representing 13% of the total US population, and Latin, 1%, even though they account for 18% of the US population (5).

In this way, the COVID-19 pandemic was reminiscent of the early days of the HIV epidemic, with poor inclusion of communities with intersectional identities in prevention and curative treatment trials and persistent failures to address these determinants of health (6). Few studies analyze sex or gender unless it is the primary focus (7), and the intersection of sex and gender with exposure to gender-affirming therapy and cultural impacts linked to gender remains poorly studied (8). Furthermore, disparities persist in including individuals from racial and ethnic backgrounds with these intersecting identities in early-phase HIV vaccine clinical trials (9).

Mindful of these findings, the National Institute of Allergy and Infectious Diseases (NIAID) quickly formed the COVID-19 Prevention Network (CoVPN), comprising the HIV Vaccine Trials Network (HVTN), the HIV Prevention Trials Network (HPTN), the AIDS Clinical Trials Group, and the Infectious Disease Clinical Research Consortium (IDCRC), to develop and assess novel COVID-19 vaccines in early 2020 (10, 11). Accelerated review of grants, contracts, and agreements was followed with a common Data and Safety Monitoring Board providing safety oversight across all US Government-funded COVID-19 vaccine trials (12). Among the goals of the CoVPN was clear accountability regarding how to maintain diversity and inclusion of participants from historically marginalized areas, with a specific focus on settings other than optimal urban settings where clinical research has traditionally occurred (13–15).

Given that our site is in an academic medical center and has been enrolling participants in HIV vaccine trials for over two decades, we evaluated our enrollment metrics to determine if the COVID-19 vaccine trials conducted at our site had similar representation as compared to contemporaneous HIV vaccine candidate trials. This study analyzed enrollment data for preventative HIV and COVID-19 vaccine trials from 2020 through 2021. Considering our history of outreach and engagement with marginalized communities for HVTN studies, we hypothesized that the CoVPN studies would have enrollment diversity analogous to HVTN studies.

## Methods

### Study design

This is a single-center retrospective study of healthy adults screened and enrolled at Brigham and Women’s Hospital in Boston, Massachusetts, between July 2020 and December 2021. Participants were included if they enrolled in a COVID-19 or HIV candidate vaccine trial; participants were excluded if they transferred from other clinical trial sites.

### Study population

Adult participants aged 18 years or older at the time of enrolling in the vaccine clinical trials, with a valid address for linkage to population-level data, were included in this study.

A total of 10 studies were selected to determine if we are accessing different communities through previous outreach and engagement efforts, with five in Phase I, one in Phase I/II, one in Phase II, one in Phase II/III, and two in Phase III (Table 1).

**Table 1:** Summary of the vaccine clinical trials included in this study.

Study number	Sponsor	NCT number	Vaccine candidate target	Phase (I, II, III, IV)
IAVI C100*	International AIDS Vaccine Initiative	NCT04173819	HIV	Phase I/II
IAVI C102	International AIDS Vaccine Initiative	NCT04794218	Lassa virus	Phase I
WHV 138	Worcester HIV Vaccine	NCT04927585	HIV	Phase Ib
HVTN 115	National Institute of Allergy and Infectious Diseases (NIAID)	NCT03220724	HIV	Phase I
HVTN 136/HPTN 092*	National Institute of Allergy and Infectious Diseases (NIAID)	NCT04212091	HIV	Phase I
HVTN 300	National Institute of Allergy and Infectious Diseases (NIAID)	NCT04915768	HIV	Phase I
mRNA-1273-P301	ModernaTX, Inc.	NCT04470427	SARS-CoV-2	Phase III
mRNA-1273-P205	ModernaTX, Inc.	NCT04927065	SARS-CoV-2	Phase II/III
VAC31518COV3001	Janssen Vaccines & Prevention B.V.	NCT04505722	SARS-CoV-2	Phase III
VAT00002	Sanofi Pasteur	NCT04762680	SARS-CoV-2	Phase II

*Denotes monoclonal antibodies.

### Data collection and management

Variables collected included age, race, ethnicity, ZIP code, and sex assigned at birth from case report forms (CRF) as self-reported by the participants. Sexual orientation, highest level of education, occupation, and household characteristics were extracted from CRFs when available by study. All variables were extracted and tabulated in Microsoft Excel.

Race and ethnicity categorizations were defined using the US Office of Management and Budget guidelines for federal data: American Indian or Alaskan Native, Asian, Black or African American, Native Hawaiian or Other Pacific Islander, and White. Hispanic identity was either recorded as a racial category or as ethnicity (16). To reconcile differences between studies and acknowledge the diversity within the participants’ identities, we recorded all participants who did not identify as Latin or Hispanic separately in a subgroup from those of Hispanic or Latin descent. Participants who selected two races or more were recorded as “Multiracial.”

### Statistical analysis

Descriptive summary statistics of participants’ demographic characteristics were calculated. Vaccine trial participants were geocoded to their census block group by linkage to their residential address. Incomplete and out-of-state addresses were excluded. Address data were obtained from metropolitan areas in Massachusetts exclusively and matched to the reported median household income level from the 2020 American Community Survey (ACS) 5-Year Estimates to account for demographic changes over time and because they are based on larger sample size, and thus more reliable, than the 1- and 3-year ACS estimates (17). These were then analyzed by frequency and quartile. Spatial analyses were performed using ArcGIS Online (https://www.arcgis.com/index.html, accessed 10 July, 2022).

## Results

**Figure 1:**
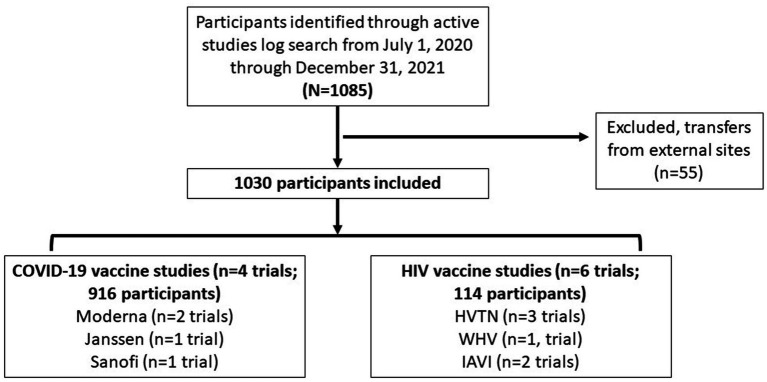
Consort diagram describing the process of how many participants in the vaccine clinical trials were eligible for inclusion.

For the study period, four COVID-19 vaccine clinical trials (*n* = 916 total participants) and six HIV vaccine trials (*n* = 114 total participants) were included (Figure 1). In the COVID-19 vaccine clinical studies, the age of participants ranged from 18 to 92 years [median = 49; interquartile range (IQR) = 33–63 years; Table 2]. About 44% of participants identified as female. Most identified as White (65.3%), and those identifying as Black/African American (7.6%) were present at slightly higher numbers than Multiracial (5.6%). Participants identifying as Hispanic compromised 24.1% of all participants.

**Table 2:** Race, ethnicity, age, and gender representation in the vaccine clinical trials.

		COVID-19 vaccine clinical trial participants (*n* = 916)	HIV vaccine clinical trial participants (*n* = 114)
Median age, years (IQR)		49 (33–63)	28 (24–34)
Sex at birth, No. (%)	Female	403 (44.0)	66 (58.0)
Race, No. (%)	White	598 (65.3)	83 (72.8)
	Black/African American	70 (7.6)	7 (6.1)
	Asian	114 (12.4)	12 (10.5)
	American Indian/Alaskan Native	4 (0.4)	2 (1.8)
	Native Hawaiian/Other Pacific Islander	3 (0.3)	0 (0)
	Multiracial	51 (5.6)	6 (5.3)
	Unknown	15 (1.6)	0 (0)
	Not reported	13 (1.4)	0 (0)
	Other	48 (5.2)	4(3.5)
Ethnicity, No. (%)	Not Hispanic or Latin	691 (75.4)	99 (86.8)
	Hispanic or Latin	221 (24.1)	15 (13.2)
	Unknown	3 (0.3)	0
	Not reported	1 (0.1)	0

The candidate HIV vaccine trial participants’ demographic characteristics are summarized in Table 2. Studies selected with kindred eligibility, follow-up, and operational features were Worcester HIV Vaccine (WHV), International AIDS Vaccine Initiative (IAVI), and HVTN studies, and two, namely IAVI C100 and HVTN 136/HPTN 092, were monoclonal antibody trials. The age of participants ranged from 18 to 50 years (median = 28; IQR = 24–34 years). A higher percentage of participants identified as female (58%), White (72.8%), and Not Hispanic (86.8%) compared to the COVID-19 vaccine clinical studies. Black/African American-identifying participants and Multiracial participants were represented at a similar frequency.

Other covariate data collected during screening and enrollment for some HIV vaccine studies were educational attainment and sexual orientation (Table 3). Out of 79 participants, only five (6.3%) had not accessed higher education. About 77% attained an undergraduate degree; 38% had at least some graduate schooling. In contrast, according to the ACS, of people living in Massachusetts, approximately 4.2% possessed middle school graduation as their highest educational attainment, 28.2% possessed some high school or high school graduation, 15.3% some college with no degree, 7.7% an associate degree, 24.5% a bachelor’s degree, and 20% a graduate or professional degree. Regarding sexual orientation in our studies, 57% identified as part of the lesbian, gay, bisexual, transgender, queer, and more (LGBTQ+) community.

**Table 3:** Educational attainment and sexual orientation for HIV vaccine studies conducted at Brigham and Women’s Hospital.

		HIV vaccine clinical trial participants (*n* = 79)
Education	Did not graduate from high school	1 (1.3)
	High school graduate or GED	4 (5.1)
	Some college/AA degree/technical school training	13(16.5)
	Undergraduate college degree (BS/BA)	31(39.2)
	Some graduate school	6 (7.6)
	Master’s degree	15 (19.0)
	Doctorate/medical degree/law degree	9 (11.4)
Sexual orientation	Gay/Lesbian/Homosexual	17 (21.5)
	Bisexual	16 (20.3)
	Queer	8 (10.1)
	Two spirit	0 (0)
	Straight/Heterosexual	33 (41.8)
	Additional category	4 (5.1)
	Not sure	1 (1.3)

**Figure 2:**
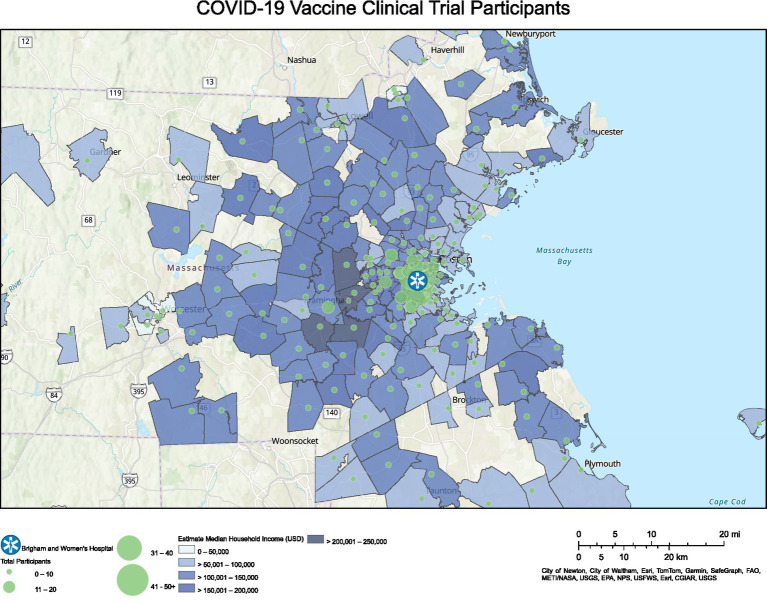
Geographical distribution for COVID-19 vaccine clinical trial participants and associated median household income.

**Figure 3:**
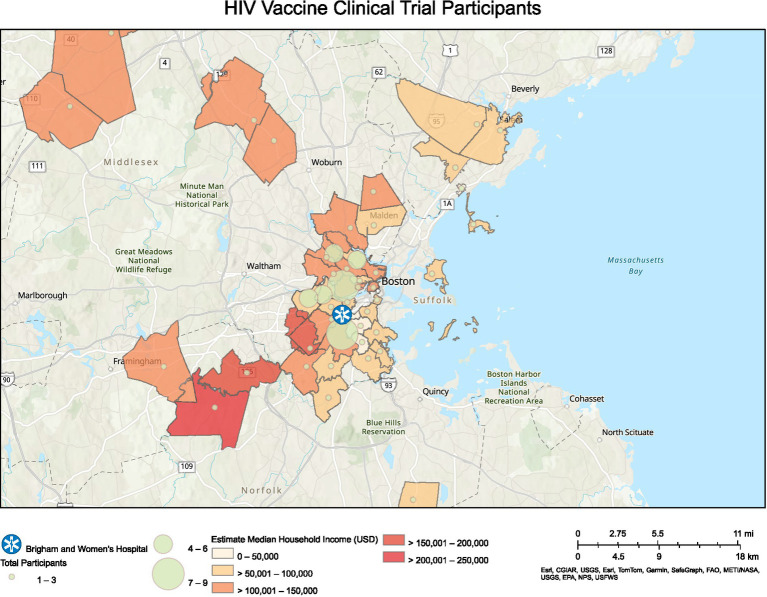
Geographical distribution for HIV vaccine clinical trial participants and associated median household income.

Subsequently, we mapped participants’ ZIP codes of residence to visually show the geographical range of participants and the density of participants living within commuting distance of our center (Figures 2, 3). Figure 2 corresponds to the COVID-19 vaccine clinical trials participants, and Figure 3 corresponds to the HIV vaccine clinical trial participants. Participants for the COVID-19 vaccine trials represented a wider geographical spread as compared to participants in the HIV vaccine trials, although there were also more participants in the COVID-19 vaccine trials.

**Figure 4:**
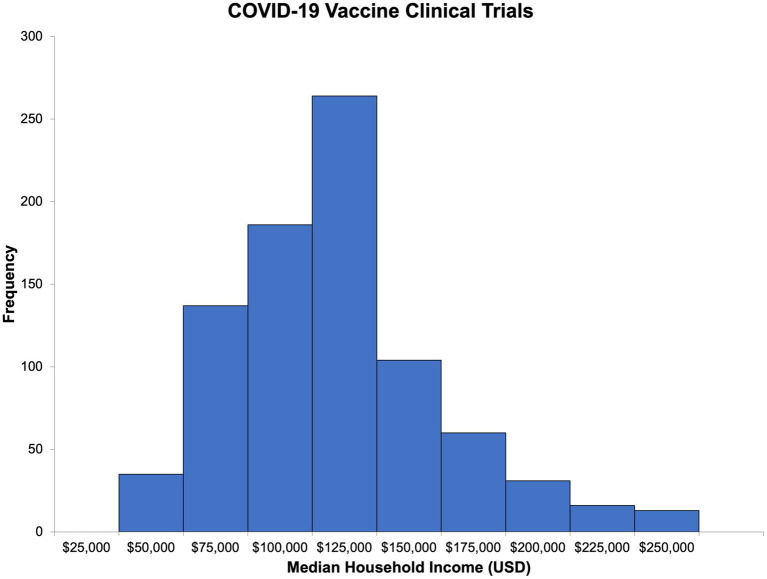
Estimated median household income for COVID-19 vaccine clinical trial participants.

**Figure 5:**
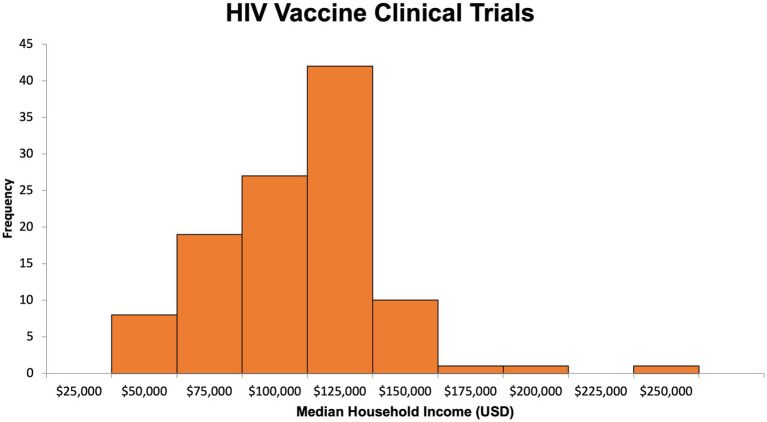
Estimated median household income for HIV vaccine clinical trial participants.

Using publicly available census-level ZIP code income data for Massachusetts residents, we were able to estimate the median household income and its distribution for participants in both types of trials (Figures 4, 5). The median household income for the ZIP code reported by COVID-19 vaccine clinical trial participants (*n* = 846) was 102,088 USD (IQR = 81,442–126,094, range 25,077–250,000; Supplementary Table S1). For HIV vaccine clinical trial participants (*n* = 109), the median household income of the reported ZIP code was 101,266 USD (IQR = 75,052–108,832, range 25,077–250,000 USD; Supplementary Table S2). Comparatively, in 2020, the median household income for Massachusetts residents was 84,385 USD, slightly lower than that of HIV and COVID-19 vaccine clinical trial participants, and interestingly, the same ZIP code, 02130, was the most commonly reported among participants for both.

## Discussion

Our study characterizes demographics of participants enrolled at our site in COVID-19 and HIV candidate vaccine trials during the peak of the COVID-19 pandemic, accentuating the enrollment characteristics of racial/ethnic minorities, females, and older individuals in vaccine clinical trials. The use of demographic characteristics to assess the success of community engagement practices on enrollment revealed that the local communities that participated in COVID-19 and HIV vaccine clinical trials appear to be only minimally different.

We found that the HIV vaccine trials enrolled a high percentage of individuals from the LGBTQ+ community. HIV itself may be selectively motivating, and the HVTN and our clinical site has longstanding dedicated initiatives and outreach to these communities, which likely influenced the success of recruiting a sexual orientation and gender-diverse community at risk. Consistent with these findings, other research has indicated that sexual minority people are more likely to utilize some health services, such as those focused on HIV prevention, than heterosexual people (18).

Of great concern, however, is the lower research participation among Black and Latin sexual minority men (18, 19). Startlingly, among many racial/ethnic minorities, awareness of trials is a significant barrier to clinical trial participation, with many reporting they are simply not asked to participate (20), and longstanding bias compounded by providers’ perception of underrepresented populations’ reluctance can lower the likelihood that a provider offer trials as a treatment option (21). Other studies have shown that undocumented immigrants often experience additional immigration-related barriers and low healthcare utilization that may limit trial participation, and immigration status measured inconsistently through the use of proxy measures (22, 23).

The findings from this study align with broader literature that has demonstrated underrepresentation in clinical research is associated with low levels of education and inadequacy in health literacy. Moreover, our study identified a disproportionate enrollment compared to the Massachusetts average with respect to socioeconomic status. Similar to outcomes reported in other scholarly work, we found that individuals who had higher levels of education (some college/completed college/graduate school) were more likely to have actively participated in a clinical trial (24).

With 7.2% of the total participants commuting from Jamaica Plain, a neighborhood of Boston in Suffolk County, Massachusetts, adjacent to our academic medical center, commuting distance may have facilitated their enrollment. Surprisingly, Jamaica Plain’s corresponding ZIP code had a median household income of 102,088 USD in 2020, the same as the median household income for COVID-19 vaccine clinical trial participants. Overall, the estimated median household income was higher for the ZIP codes reported by COVID-19 vaccine clinical trial participants, which may be due to the higher age range of the participants. In the HIV vaccine studies, volunteers could only be enrolled within the age range of 18–50 years rather than 18 years and older, as in the COVID-19 vaccine studies, suggesting this finding was due to eligibility criteria and study design, not failure in our recruitment efforts.

Albeit individual-level income, a known social determinant of health, was not collected in our studies, using ZIP code-level median household income as a surrogate to approximate socioeconomic status can aid in identifying inequities in access, socioeconomic barriers, implicit bias, and outcomes when no direct income data is available (25). In future studies, we propose that income ranges could be placed in intake forms at the time of enrollment to allow a more detailed analysis of socioeconomic status in recruitment/retention and maps overlaying high deprivation index and high diversity areas with existing hospitals, existing major HIV trial centers, and commuting distance to the closest HIV trial center to identify biases in the sociodemographic of populations living within commuting distance.

Apart from providing a detailed reference for our single center, we hope our work will encourage further collaborative efforts with nearby hospitals, community clinics, and community centers closer to underrepresented populations to lessen the socioeconomic burdens of clinical trial participation. Rather than allowing rapid digitization without considering community engagement processes, resulting in deepening health inequities, implementing mobile data collection, online recruitment, and strategic approaches for those traditionally hard-to-reach can more broadly engage these communities (26–28). After all, providing community benefits will strengthen community capacity and agency.

Our data suggest a need to address not only the difficulties that come with categorized race reporting but the urgent need to harmonize data collection methods. Inferring racial makeup using definitions that do not capture the increasing diversity of the US population calls for updated definitions, as there is no consensus about how to define race and ethnicity (29, 30). Shifting demographics demonstrate that non-white US populations are projected to become a majority population by 2044. Determining the optimal way to record race and ethnicity data when compiling descriptive statistics is vital in improving trials’ precision and equitable application in practice.

There are a few limitations identified in this study. First, the data available were limited due to the retrospective design and, therefore, relied on data captured within the participants’ clinical trial documentation and electronic health records. Another concern is that self-reported race/ethnicity was inconsistent across trials, particularly for Hispanic and Multiracial populations, and thus, estimates of Hispanic enrollment may be inaccurate. Third, individual-level data concerning socioeconomic status are absent; thus, ZIP codes and associated median incomes are an approximation. We attempted to account for this with the use of aggregate-level data. Finally, this study was conducted at a single academic medical center in an urban setting. Therefore, the generalizability of the findings may be limited, and future research is warranted within non-urban settings and across multiple centers.

## Conclusion

In this retrospective study, we describe the characteristics of participants enrolled for HIV and COVID-19 vaccine trials at a single center in Boston, Massachusetts. We describe similarities in geographical distribution, median incomes, and proportion of underrepresented individuals between the two types of trials. We found that the HIV vaccine trials successfully recruited individuals from sexual orientation and gender-diverse backgrounds. Our findings demonstrate that further outreach efforts are needed to ensure the inclusion of individuals from lower educational and socioeconomic brackets. In addition, continued and sustained efforts are needed to ensure inclusion of individuals from diverse racial and ethnic backgrounds. We illustrate the importance, benefits, and contribution of sustained community relationships to provide direction and evidence-based support for the inclusion of historically marginalized subgroups in clinical trials of vaccines for COVID-19, HIV, and other emerging pathogens.

## Data availability statement

The data analyzed in this study is subject to the following licenses/restrictions: Confidential data that includes potentially identifiable data. Requests to access these datasets should be directed to acsherman@bwh.harvard.edu.

## Ethics statement

The studies involving humans were approved by Brigham and Women’s Institutional Review Board. The studies were conducted in accordance with the local legislation and institutional requirements. Written informed consent for participation was not required from the participants or the participants’ legal guardians/next of kin because IRB determined informed consent was not required since there was no intervention or interaction performed. This was a retrospective review only.

## Author contributions

DL: Conceptualization, Data curation, Formal analysis, Investigation, Methodology, Validation, Writing – original draft, Writing – review & editing. EK: Data curation, Resources, Writing – review & editing. OO: Data curation, Writing – review & editing. JP: Data curation, Methodology, Writing – review & editing. UK: Data curation, Investigation, Writing – review & editing. NM: Data curation, Investigation, Writing – review & editing. AA: Data curation, Investigation, Writing – review & editing. AT: Data curation, Investigation, Writing – review & editing. LB: Resources, Supervision, Writing – review & editing. AS: Conceptualization, Data curation, Formal analysis, Investigation, Methodology, Resources, Supervision, Writing – review & editing. SW: Investigation, Methodology, Project administration, Resources, Supervision, Writing – review & editing.
