# Supplementary material for: Evaluating enrollment and representation in COVID-19 and HIV vaccine clinical trials
Source: Front Public Health. 2024 Jul 26;12:1411970. doi: 10.3389/fpubh.2024.1411970 (PMC11311253; doi:10.3389/fpubh.2024.1411970)
Supplement: Supplementary file 1 [file Table_1.DOCX]

**Supplemental Table 1.** Estimate median household income in the COVID-19 vaccine clinical trials.

|  | **ZIP Code** | **No. (%) of participants** | **Estimate Median Household Income (USD)** |
| --- | --- | --- | --- |
| **Median (IQR)** |  |  | $102,088 ($81,442-$126,094) |
| **COVID-19 Vaccine Clinical Trial Participants (n=846)** | 02130 | 52 (6.1) | $102,088 |
|  | 02446 | 35 (4.1) | $99,383 |
|  | 02135 | 20 (2.4) | $83,383 |
|  | 02445 | 20 (2.4) | $126,094 |
|  | 02139 | 19 (2.2) | $108,832 |
|  | 02138 | 17 (2.0) | $104,641 |
|  | 02116 | 16 (1.9) | $115,825 |
|  | 02215 | 14 (1.7) | $55,468 |
|  | 02131 | 14 (1.7) | $87,083 |
|  | 02134 | 13 (1.5) | $66,095 |
|  | 02118 | 13 (1.5) | $62,850 |
|  | 02114 | 13 (1.5) | $107,162 |
|  | 02459 | 12 (1.4) | $200,091 |
|  | 02467 | 11 (1.3) | $150,223 |
|  | 02478 | 11 (1.3) | $140,091 |
|  | 02115 | 11 (1.3) | $45,827 |
|  | 01760 | 11 (1.3) | $115,588 |
|  | 02474 | 10 (1.2) | $115,000 |
|  | 02493 | 10 (1.2) | $206,250 |
|  | 01742 | 9 (1.1) | $160,448 |
|  | 02155 | 8 (0.9) | $101,266 |
|  | 02150 | 8 (0.9) | $60,305 |
|  | 02332 | 8 (0.9) | $126,889 |
|  | 02136 | 8 (0.9) | $76,704 |
|  | 02140 | 8 (0.9) | $114,256 |
|  | 02128 | 7 (0.8) | $63,378 |
|  | 02111 | 7 (0.8) | $52,663 |
|  | 02186 | 7 (0.8) | $141,050 |
|  | 02169 | 7 (0.8) | $76,636 |
|  | 02132 | 7 (0.8) | $117,083 |
|  | 02472 | 7 (0.8) | $100,399 |
|  | 02125 | 6 (0.7) | $60,862 |
|  | 02129 | 6 (0.7) | $131,064 |
|  | 01776 | 6 (0.7) | $195,073 |
|  | 02124 | 6 (0.7) | $63,118 |
|  | 02210 | 6 (0.7) | $170,920 |
|  | 02492 | 6 (0.7) | $184,688 |
|  | 02144 | 6 (0.7) | $111,549 |
|  | 02143 | 6 (0.7) | $105,193 |
|  | 02494 | 6 (0.7) | $139,770 |
|  | 02145 | 6 (0.7) | $90,491 |
|  | 02461 | 5 (0.6) | $171,855 |
|  | 02090 | 5 (0.6) | $159,776 |
|  | 02468 | 5 (0.6) | $250,000 |
|  | 02180 | 5 (0.6) | $103,104 |
|  | 02170 | 5 (0.6) | $85,821 |
|  | 01970 | 5 (0.6) | $66,495 |
|  | 02126 | 5 (0.6) | $55,126 |
|  | 02148 | 5 (0.6) | $73,399 |
|  | 01960 | 5 (0.6) | $80,679 |
|  | 02141 | 5 (0.6) | $87,739 |
|  | 01852 | 5 (0.6) | $65,445 |
|  | 02188 | 5 (0.6) | $93,160 |
|  | 02481 | 5 (0.6) | $227,898 |
|  | 02142 | 5 (0.6) | $134,575 |
|  | 01702 | 5 (0.6) | $67,048 |
|  | 02119 | 5 (0.6) | $32,424 |
|  | 02176 | 4 (0.5) | $114,604 |
|  | 02171 | 4 (0.5) | $85,586 |
|  | 02025 | 4 (0.5) | $141,036 |
|  | 01810 | 4 (0.5) | $153,315 |
|  | 01843 | 4 (0.5) | $54,283 |
|  | 02053 | 4 (0.5) | $139,688 |
|  | 01945 | 4 (0.5) | $131,293 |
|  | 02120 | 4 (0.5) | $46,843 |
|  | 02368 | 4 (0.5) | $87,803 |
|  | 01887 | 4 (0.5) | $133,873 |
|  | 02451 | 4 (0.5) | $105,536 |
|  | 01801 | 4 (0.5) | $92,084 |
|  | 01906 | 4 (0.5) | $88,348 |
|  | 01581 | 4 (0.5) | $119,148 |
|  | 02048 | 4 (0.5) | $120,694 |
|  | 02026 | 3 (0.4) | $101,730 |
|  | 01867 | 3 (0.4) | $131,515 |
|  | 02359 | 3 (0.4) | $119,827 |
|  | 02465 | 3 (0.4) | $157,449 |
|  | 02127 | 3 (0.4) | $122,599 |
|  | 01778 | 3 (0.4) | $192,961 |
|  | 02149 | 3 (0.4) | $70,627 |
|  | 01841 | 3 (0.4) | $45,486 |
|  | 02703 | 3 (0.4) | $77,107 |
|  | 02062 | 3 (0.4) | $90,341 |
|  | 02121 | 3 (0.4) | $36,479 |
|  | 02030 | 3 (0.4) | $250,000 |
|  | 01719 | 3 (0.4) | $126,597 |
|  | 01832 | 3 (0.4) | $67,016 |
|  | 02740 | 3 (0.4) | $47,514 |
|  | 01923 | 3 (0.4) | $99,269 |
|  | 02453 | 3 (0.4) | $87,922 |
|  | 02466 | 3 (0.4) | $118,333 |
|  | 01770 | 3 (0.4) | $216,406 |
|  | 02184 | 3 (0.4) | $101,544 |
|  | 02113 | 2 (0.2) | $97,686 |
|  | 02152 | 2 (0.2) | $76,996 |
|  | 01532 | 2 (0.2) | $140,600 |
|  | 02021 | 2 (0.2) | $107,442 |
|  | 01890 | 2 (0.2) | $173,058 |
|  | 01826 | 2 (0.2) | $92,533 |
|  | 02081 | 2 (0.2) | $124,528 |
|  | 02420 | 2 (0.2) | $198,110 |
|  | 01905 | 2 (0.2) | $62,623 |
|  | 02035 | 2 (0.2) | $92,978 |
|  | 01880 | 2 (0.2) | $104,609 |
|  | 02766 | 2 (0.2) | $108,435 |
|  | 01701 | 2 (0.2) | $116,971 |
|  | 02190 | 2 (0.2) | $87,424 |
|  | 01938 | 2 (0.2) | $103,941 |
|  | 01904 | 2 (0.2) | $87,639 |
|  | 02301 | 2 (0.2) | $57,977 |
|  | 02458 | 2 (0.2) | $120,385 |
|  | 01773 | 2 (0.2) | $156,607 |
|  | 01915 | 2 (0.2) | $84,354 |
|  | 02482 | 2 (0.2) | $201,548 |
|  | 01772 | 2 (0.2) | $163,438 |
|  | 02109 | 2 (0.2) | $156,119 |
|  | 02452 | 2 (0.2) | $99,747 |
|  | 02122 | 2 (0.2) | $72,158 |
|  | 01824 | 2 (0.2) | $123,087 |
|  | 02052 | 2 (0.2) | $174,417 |
|  | 02568 | 2 (0.2) | $52,711 |
|  | 02720 | 2 (0.2) | $52,753 |
|  | 02151 | 2 (0.2) | $68,199 |
|  | 01879 | 2 (0.2) | $114,313 |
|  | 02339 | 2 (0.2) | $133,922 |
|  | 02038 | 2 (0.2) | $118,193 |
|  | 01749 | 2 (0.2) | $96,038 |
|  | 01902 | 2 (0.2) | $52,834 |
|  | 01940 | 2 (0.2) | $136,023 |
|  | 01748 | 2 (0.2) | $172,683 |
|  | 01930 | 2 (0.2) | $76,260 |
|  | 02460 | 2 (0.2) | $126,185 |
|  | 01545 | 2 (0.2) | $108,870 |
|  | 02108 | 2 (0.2) | $152,442 |
|  | 02633 | 2 (0.2) | $81,696 |
|  | 02067 | 2 (0.2) | $144,280 |
|  | 02760 | 2 (0.2) | $91,592 |
|  | 02043 | 2 (0.2) | $147,520 |
|  | 01524 | 2 (0.2) | $80,649 |
|  | 01845 | 2 (0.2) | $113,916 |
|  | 02346 | 2 (0.2) | $77,455 |
|  | 01886 | 2 (0.2) | $149,437 |
|  | 01564 | 1 (0.1) | $122,810 |
|  | 01741 | 1 (0.1) | $197,530 |
|  | 02072 | 1 (0.1) | $94,823 |
|  | 01515 | 1 (0.1) | $75,347 |
|  | 01950 | 1 (0.1) | $110,740 |
|  | 01062 | 1 (0.1) | $76,959 |
|  | 01450 | 1 (0.1) | $169,643 |
|  | 01720 | 1 (0.1) | $138,750 |
|  | 01603 | 1 (0.1) | $45,969 |
|  | 01608 | 1 (0.1) | $31,504 |
|  | 02556 | 1 (0.1) | $100,543 |
|  | 02382 | 1 (0.1) | $88,932 |
|  | 02056 | 1 (0.1) | $159,914 |
|  | 01451 | 1 (0.1) | $177,422 |
|  | 01863 | 1 (0.1) | $112,568 |
|  | 01503 | 1 (0.1) | $104,432 |
|  | 02364 | 1 (0.1) | $95,197 |
|  | 01339 | 1 (0.1) | $60,438 |
|  | 02061 | 1 (0.1) | $162,091 |
|  | 01754 | 1 (0.1) | $107,891 |
|  | 01840 | 1 (0.1) | $25,077 |
|  | 02054 | 1 (0.1) | $114,255 |
|  | 01060 | 1 (0.1) | $67,356 |
|  | 02557 | 1 (0.1) | $90,313 |
|  | 02657 | 1 (0.1) | $71,495 |
|  | 02725 | 1 (0.1) | $98,459 |
|  | 01609 | 1 (0.1) | $45,679 |
|  | 02779 | 1 (0.1) | $106,515 |
|  | 01082 | 1 (0.1) | $58,131 |
|  | 02790 | 1 (0.1) | $76,134 |
|  | 02464 | 1 (0.1) | $123,098 |
|  | 01746 | 1 (0.1) | $137,589 |
|  | 01803 | 1 (0.1) | $121,433 |
|  | 01951 | 1 (0.1) | $115,719 |
|  | 02537 | 1 (0.1) | $114,737 |
|  | 02343 | 1 (0.1) | $79,718 |
|  | 02163 | 1 (0.1) | $57,989 |
|  | 01821 | 1 (0.1) | $116,445 |
|  | 02066 | 1 (0.1) | $127,646 |
|  | 02780 | 1 (0.1) | $59,663 |
|  | 02351 | 1 (0.1) | $106,001 |
|  | 01344 | 1 (0.1) | $63,333 |
|  | 01983 | 1 (0.1) | $144,258 |
|  | 01854 | 1 (0.1) | $55,461 |
|  | 01944 | 1 (0.1) | $178,250 |
|  | 02543 | 1 (0.1) | $82,857 |
|  | 01901 | 1 (0.1) | $26,403 |
|  | 01462 | 1 (0.1) | $99,601 |
|  | 01860 | 1 (0.1) | $79,909 |
|  | 01876 | 1 (0.1) | $105,480 |
|  | 02476 | 1 (0.1) | $113,978 |
|  | 01605 | 1 (0.1) | $42,683 |
|  | 01721 | 1 (0.1) | $118,348 |
|  | 02653 | 1 (0.1) | $77,318 |
|  | 02671 | 1 (0.1) | $78,266 |
|  | 01590 | 1 (0.1) | $108,971 |
|  | 01864 | 1 (0.1) | $123,042 |
|  | 02360 | 1 (0.1) | $92,757 |
|  | 01833 | 1 (0.1) | $122,600 |
|  | 01516 | 1 (0.1) | $117,250 |
|  | 01607 | 1 (0.1) | $53,838 |
|  | 02767 | 1 (0.1) | $107,297 |
|  | 01440 | 1 (0.1) | $51,708 |
|  | 01850 | 1 (0.1) | $55,837 |
|  | 01523 | 1 (0.1) | $97,394 |
|  | 01752 | 1 (0.1) | $83,469 |
|  | 02330 | 1 (0.1) | $61,374 |
|  | 01569 | 1 (0.1) | $109,179 |
|  | 01730 | 1 (0.1) | $133,824 |
|  | 01331 | 1 (0.1) | $61,949 |
|  | 02747 | 1 (0.1) | $83,459 |
|  | 02535 | 1 (0.1) | $98,354 |
|  | 01536 | 1 (0.1) | $102,756 |
|  | 02649 | 1 (0.1) | $78,309 |
|  | 01602 | 1 (0.1) | $71,239 |
