# Supplementary material for: Evaluating enrollment and representation in COVID-19 and HIV vaccine clinical trials
Source: Front Public Health. 2024 Jul 26;12:1411970. doi: 10.3389/fpubh.2024.1411970 (PMC11311253; doi:10.3389/fpubh.2024.1411970)
Supplement: Supplementary file 2 [file Table_2.DOCX]

**Supplemental Table 2.** Estimate median household income in the HIV vaccine clinical trials.

|  | **ZIP Code** | **No. (%) of participants** | **Estimate Median Household Income (USD)** |
| --- | --- | --- | --- |
| **Median (IQR)** |  |  | $101,266 ($75,052- $108,832) |
| **HIV Vaccine Clinical Trial Participants (n=109)** | 02130 | 9 (8.3) | $102,088 |
|  | 02139 | 8 (7.3) | $108,832 |
|  | 02135 | 6 (5.5) | $83,383 |
|  | 02145 | 6 (5.5) | $90,491 |
|  | 02134 | 6 (5.5) | $66,095 |
|  | 02144 | 5 (4.6) | $111,549 |
|  | 02138 | 3 (2.8) | $104,641 |
|  | 02114 | 3 (2.8) | $107,162 |
|  | 02129 | 3 (2.8) | $131,064 |
|  | 02143 | 3 (2.8) | $105,193 |
|  | 02445 | 3 (2.8) | $126,094 |
|  | 02118 | 3 (2.8) | $62,850 |
|  | 02155 | 3 (2.8) | $101,266 |
|  | 02119 | 2 (1.8) | $32,424 |
|  | 02124 | 2 (1.8) | $63,118 |
|  | 02141 | 2 (1.8) | $87,739 |
|  | 02125 | 2 (1.8) | $60,862 |
|  | 02142 | 2 (1.8) | $134,575 |
|  | 02446 | 2 (1.8) | $99,383 |
|  | 02140 | 2 (1.8) | $114,256 |
|  | 02215 | 2 (1.8) | $55,468 |
|  | 02120 | 2 (1.8) | $46,843 |
|  | 02121 | 2 (1.8) | $36,479 |
|  | 01960 | 1 (0.9) | $80,679 |
|  | 01886 | 1 (0.9) | $149,437 |
|  | 01821 | 1 (0.9) | $116,445 |
|  | 01840 | 1 (0.9) | $25,077 |
|  | 01913 | 1 (0.9) | $81,027 |
|  | 02122 | 1 (0.9) | $72,158 |
|  | 01901 | 1 (0.9) | $26,403 |
|  | 02030 | 1 (0.9) | $250,000 |
|  | 01760 | 1 (0.9) | $115,588 |
|  | 02343 | 1 (0.9) | $79,718 |
|  | 02148 | 1 (0.9) | $73,399 |
|  | 02136 | 1 (0.9) | $76,704 |
|  | 02360 | 1 (0.9) | $92,757 |
|  | 02492 | 1 (0.9) | $184,688 |
|  | 01803 | 1 (0.9) | $121,433 |
|  | 01908 | 1 (0.9) | $94,243 |
|  | 02111 | 1 (0.9) | $52,663 |
|  | 02132 | 1 (0.9) | $117,083 |
|  | 01460 | 1 (0.9) | $125,275 |
|  | 02668 | 1 (0.9) | $96,165 |
|  | 01863 | 1 (0.9) | $112,568 |
|  | 02131 | 1 (0.9) | $87,083 |
|  | 02467 | 1 (0.9) | $150,223 |
|  | 01904 | 1 (0.9) | $87,639 |
|  | 01970 | 1 (0.9) | $66,495 |
|  | 02675 | 1 (0.9) | $84,404 |
|  | 02152 | 1 (0.9) | $76,996 |
|  | 02176 | 1 (0.9) | $114,604 |
